# Integrating rheumatology care in the community: can shared care work?

**DOI:** 10.5334/ijic.1990

**Published:** 2015-08-19

**Authors:** Anita YN Lim, Chuen Seng Tan, Bernadette PL Low, Tang Ching Lau, Tze Lee Tan, Lee Gan Goh, Gim Gee Teng

**Affiliations:** Division of Rheumatology, University Medicine Cluster, National University Health System, Singapore; Department of Medicine, Yong Loo Lin School of Medicine, National University of Singapore, Singapore; Saw Swee Hock School of Public Health, National University of Singapore, Singapore; Care Integration and Alliances, National University Health System, Singapore; Division of Rheumatology, University Medicine Cluster, National University Health System, Singapore; Department of Medicine, Yong Loo Lin School of Medicine, National University of Singapore, Singapore; Yong Loo Lin School of Medicine, National University of Singapore, Singapore; Division of Family Medicine, University Medicine Cluster, National University Health System, Institute of Family Medicine, College of Family Physicians, Singapore; Yong Loo Lin School of Medicine, National University of Singapore, Singapore; Institute of Family Medicine, College of Family Physicians, Singapore; Division of Rheumatology, University Medicine Cluster, National University Health System, Singapore; Department of Medicine, Yong Loo Lin School of Medicine, National University of Singapore, Singapore

**Keywords:** musculoskeletal diseases, rheumatology, family physician, integrated care, financing, Singapore

## Abstract

**Introduction:**

Singapore's rapidly ageing population and chronic disease burden at public hospital specialist clinics herald a silver tsunami. In Singapore, “right siting” aims to manage stable chronic disease in primary care at a lower cost. To improve the quality of rheumatology care, we created shared care between rheumatologist and family physician to reduce hospital visits.

**Methods:**

Clinical practice improvement methodology was used to structure shared care of stable patients between hospital rheumatologists and eleven community family physicians; one ran a hospital clinic. A case manager coordinated the workflow.

**Results:**

About 220 patients entered shared care over 29 months. Patients without hospital subsidies (private patients) and private family physicians independently predicted successful shared care, defined as one cycle of alternating care.

**Discussion:**

Our shared care model incorporated a case manager, systematic workflows, patient selection criteria, willing family physician partners and rheumatologists in the absence of organizational integration. Health care affordability impacts successful shared care. Government subsidy hindered right siting to private primary care.

**Conclusions:**

Financing systems in Singapore, at health policy level, must allow transfer of hospital subsidies to primary care, both private and public, to make it more affordable than hospital care. Structural integration will create a seamless continuum between hospital and primary care.

## Introduction

Musculoskeletal disorders rank highest in prevalence as the causes of chronic ill health, long-term disabilities and consultation with health professionals, and rank second for restricted activity days and use of prescription and non-prescription drugs [1–3]. The Singapore Burden of Disease Study 2010 found that musculoskeletal diseases was the fifth leading cause of disability (6% of disease burden), similar to that in developed regions of the world [4]. Singapore's rapidly ageing population and changing disease profiles call for urgent implementation of community-based chronic disease care delivery to provide comprehensive, accessible, affordable, quality care [5].

## Problem statement

The management of musculoskeletal problems relies heavily on referral to tertiary care in the Singapore health care system. These include self-limiting problems such as soft tissue complaints, chronic conditions such as gout and osteoarthritis, which require an initial consultation for diagnosis and management plan and return to primary care. Patients with musculoskeletal diseases which include inflammatory arthritis, however, require close monitoring and frequent hospital appointments. Laboratory monitoring for toxicities associated with immunosuppressant therapy mandates regular hospital review as there is no defined primary care physician for each individual in Singapore. This system of follow-up can account for approximately 75% of the rheumatologist's workload [6]. Rheumatologists’ time could be better utilised in assessing new patients and continuing the management of complex disease if stable patients’ care could be transitioned to primary care. Singapore has 48 rheumatologists; the majority provides care within the public sector, serving a population of 5.5 million. Limited and delayed access to the rheumatologist hinders the provision of appropriate and timely care for patients with rheumatic diseases [7]. Median waiting time for a rheumatology appointment in Singapore public hospitals is 45 days.

Singapore's health care system is dual, public and private. Unlike the British National Health Service, where residents are entitled to health care that is mostly free at the point of consumption, the Singapore government promotes personal responsibility for one's health and the avoidance of over-reliance on state welfare or medical insurance. Singapore's health care financing system is based on patient's cost-sharing or co-payment either through cash or Medisave, a mandatory medical savings programme and a low cost medical insurance scheme (Medishield). Subsidized class patients between the ages of 18 and 65 receive 50% concession; others receive 75% concession for outpatient consultations and investigations. A Standard Drug List provides a wide range of essential drugs at affordable prices in public institutions. Inpatient subsidies are even more substantial. However, patients are free to choose between public or private providers; for the latter, no government concessions are available. In the absence of supplementary private insurance plans, out of pocket costs are higher for those who choose private care. Private care is available also in public hospitals although their primary mission is to serve the disproportionately larger pool of subsidized class patients.

Singapore's total population was last recorded at 5.5 million people in 2014 from 1.7 million in 1960, changing 232% during the last 50 years. Exponential population growth over the past five years strained infrastructure and public services, prompting a surge in the need for new health care infrastructure and health care professionals. In 2013, the Singapore Registry of Births and Deaths recorded that cancer and cardiovascular diseases accounted for 60% of total deaths in Singapore as compared to about 25% of deaths in 1960. The epidemiological transition of the major causes of deaths from communicable to non-communicable diseases together with changing demographics with falling birth rates (crude birth rate per 1,000 residents in 1970 was 22.1 as compared to 10.1 in 2012) coupled with longer life expectancy at birth (65.8 years in 1970 as compared to 82.3 in 2012) have produced a silver tsunami.

Singapore's health care landscape has evolved to favour specialization and tertiary care as funding and health policy have catered to “more costly hospital care” [8] and the growth of hospitals and national specialist centres, leaving the primary care sector to free market forces. The challenge to manage chronic disease is heightened by the disparity of generalists delivering primary care in the public sector. About 86% of general practitioners work in the private sector, whereas 14% work in government health care centres (polyclinics), managing nearly half the load of chronic conditions [9].“Right siting” in the Singapore public health care system describes the principle whereby patients with stable chronic disease should be managed in primary care rather than specialist settings, at a lower cost [10]. Rising patient loads at specialist outpatient clinics in public hospitals, where traditionally patients with chronic diseases are cared for, make it an urgent priority.

Our primary objective was to improve the quality of rheumatology care by creating shared care between the rheumatologist and family physician to reduce the burden of hospital care. Our secondary objective was to actively discharge patients. Both objectives therefore fulfilled the goal of right siting. The concept of shared care was introduced to pilot a new model of care. Improving family physician skills, assigning appropriate patients to family physicians and coordinating care between family physicians and rheumatologists were used to achieve the objective. Shared care refers to care of patients where treatment is initiated in the specialist setting but, at an agreed time, prescribing and drug monitoring is shared with primary care. The rheumatologist's responsibility is to request a sharing of care and provide written guidance on the arrangements for sharing of care between the family physician and hospital specialist.

## Methods

### Clinical practice improvement method

We used clinical practice improvement methodology to look at how to increase patient numbers participating in shared care or being discharged [11]. The diagnostic journey by stakeholders (rheumatologist, case manager, family physician, rheumatology nurse) identified the main reasons for low numbers of patients being right-sited as receiving subsidized medical care in the hospital, rheumatologists’ reluctance to move patients out, lack of a standardized workflow for right siting and patients’ uncertainty of adequacy of care. Patients’ knowledge, attitudes and perception towards shared care were previously sought through a questionnaire survey in November 2009. Of the 315 patients surveyed, only 12 (3.8%) had heard of shared care and 187 (59.4%) wanted to participate. The main deterrent for participation was preference for specialist care 93 (62.4%).

### Patient population

**Figure 1.**
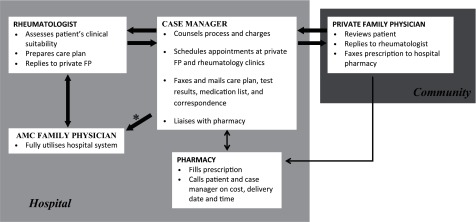
Workflow of shared care for patients with musculoskeletal diseases between rheumatologist and family physicians. AMC, academic medical centre; FP, family physician; 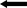, patient's journey; 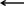, Liaison. *Reasons for refusal of shared care with private FP include personal reasons, full or substantial subsidy by hospital for low income patients, private medical insurance, employment health care benefits and government funding for civil servants.

This collaboration was between the divisions of Family Medicine and Rheumatology, of an academic medical centre, providing tertiary rheumatology care. Rheumatologists identified patients suitable for shared care and referred them to the case manager. Diagnoses included rheumatoid arthritis, psoriatic arthritis, spondyloarthritis, systemic lupus erythematosus and Sjogren's syndrome. Suitability for shared care was determined, primarily not by diagnosis, but by stability of disease activity, i.e. patients in the preceding year who had no major adjustments to their medications. Patients with osteoarthritis, gout and soft tissue complaints were discharged. Therefore, right siting incorporates both shared care and discharge to primary care (Figure 1).

### Case manager

The case manager's role was pivotal as she coordinated care processes between the patient, rheumatologist, family physician and pharmacy, collected data and monitored communication gaps between the stakeholders. We developed the patient instruction brochure which included the flow chart in order to facilitate patient understanding of the concept and processes of shared care. An individualized care plan incorporating instructions to maintain or alter drug dose and blood monitoring requirements was developed. The case manager coordinated appointments for patients with the family physician and the Rheumatology Clinic and monitored the patient's attendance. Prescriptions were tracked to ensure that pharmacy received, dispensed and delivered medication. A shared care patient instruction brochure was given to each patient whom she met at the Rheumatology Clinic. She was accessible to patients and family physicians for queries via a telephone helpline. Patient summary forms for the rheumatologist and family physician were created to standardize communication and include information on disease activity and blood test monitoring requirements.

### Family physician

11 (10 private, 1 polyclinic) family physicians practicing in the west of Singapore, the catchment area of our academic medical centre, who had minimally attained the graduate diploma in family medicine thus enabling their registration as family physicians, were identified. A team comprising a rheumatologist (AL), family physician (GLG) and case manager visited the selected family physician in his/ her clinic. Nine private family physicians agreed that their consultation charges would be equivalent to hospital Rheumatology Clinic charges. All attended our ambulatory rheumatology teaching clinics to familiarize themselves with musculoskeletal diseases. Polyclinic family physicians declined participation citing their workload except for one who had previous rheumatology training. Continuing Medical Education (six hours with attendance by 178 doctors from both public and private primary care) was conducted to publicize the shared care programme.

A weekly family medicine clinic dedicated to rheumatology, run by a private family physician was opened in the academic medical centre. This operated concurrently with Rheumatology Clinics to facilitate the smooth transition of patients as an alternative to right siting in the community. Patients whose outpatient bill reimbursement was contingent upon them remaining within the public sector, for example, those holding Singapore Civil Service Cards which meant they had no out of pocket expenses, those on medical social worker assistance and having increased government subsidies and those having private health insurance making them eligible only for claims within public hospitals, would see the academic medical centre-family physician.

### Pharmacy

As disease modifying anti-rheumatic drugs were not stocked by the family physicians whose practice included dispensing medications from their own clinics, our hospital pharmacy endorsed prescriptions from partner family physicians and arranged for medication delivery to patients for SGD2.50, hence reducing inconvenience to patients.

### Statistical analysis

220 patients (August 2010 to December 2012) progressed through the shared care cycle. One successful shared care cycle was defined as the patient completing first family physician visit followed by a Rheumatology Clinic visit and agreeing to alternating visits with the family physician. Failure included those who did not show for appointment, did not complete one cycle or chose to remain with Rheumatology Clinic or family physician. We grouped the sole polyclinic family physician together with the private family physicians in the analysis with reference to the academic medical centre-family physician. Patient characteristics and clinical variables were compared between patients who continued and ended shared care. For categorical variables, Fisher's Exact test was used to compare differences in proportions. To summarize continuous variables, we used the mean and standard deviations, and the two-sample *t*-test to compare differences in means. For skewed continuous variables, the median and inter-quartile ranges were used to summarize the variables and the Mann–Whitney test was used to compare differences in medians. We performed a modified backward variable selection on an initial multiple logistic regression model that consisted of variables with *p* value <0.1 from the univariate analysis with adjustment for confounding factors (i.e. demographic variables: age, gender and ethnicity; these variables remain in the model throughout the variable selection process regardless of *p* value). Shared care status (where 1 denotes continued shared care, and 0 denotes failed shared care) is the outcome. We removed non-demographic variables with *p* value >0.05 from the multiple logistic regression. We assessed the goodness-of-fit of the final model with the Hosmer–Lemeshow test.

We applied survival analysis to analyse the time interval between enrolment into shared care and first return to Rheumatology Clinic after seeing the family physician among 100 patients who continued shared care. Simple Cox Proportional Hazard (Cox-PH) regression model was used to determine the factors associated with the time between sending out and return to Rheumatology Clinic. The multiple Cox-PH regression model included variables with *p* values <0.1 based on the simple Cox-PH analysis with adjustment for demographic factors (age, gender and ethnicity). We applied a similar model building strategy described for the multiple logistic regression. To satisfy the proportional hazard assumption, we relaxed the criterion in the last step by allowing non-demographic variables of borderline significance (i.e. *p* value between 0.05 and 0.10) to be individually added into the Cox-PH regression model. All statistical analyses were carried out using STATA version 13.0 (StataCorp LP, College Station, TX, USA), assuming a two-sided test. *p* values <0.05 were considered significant.

## Results

A total of 6,203 patients were seen by nine rheumatology consultants and two registrars over 29 months. The majority required uninterrupted rheumatology care while 1,592 (25.7%) patients were right-sited. Of the right-sited patients, 220 (13.8%) were enrolled in shared care and 1,372 (86.2%) were discharged. Patients were discharged to polyclinics for continuing care (107), our academic medical centre-family physician (195) or given no follow-up (1,070) when no ongoing rheumatology input was required.

### Demographic and clinical characteristics

Of the 220 patients in the shared care programme, the majority were females (70.9%) and Chinese (77.7%) with a mean age of 54.7 (SD 14.9) years. This demographic profile was not significantly different from patients with rheumatic diseases seen in our clinics whose average age was 52.5 (SD 16.0) years. The median length of stay with Rheumatology Clinic was 2.42 years (inter-quartile range 0.84–4.70 years). Most were subsidized class patients (89.1%) and 65.9% were sent to private family physician. Half of the patients had one disease modifying anti-rheumatic drug (49.1%). The demographic and clinical characteristics of patients with successful shared care (*n* = 100) and those who failed shared care (*n* = 120) are shown in Table 1.

### Continuing shared care

After adjusting for age, gender and ethnicity (Chinese, Malay and others), private class patients were four times more likely to continue shared care compared to subsidized class patients (odds ratio (OR) = 4.18; 95%CI: 1.49–11.74). Patients sent to the private family physician were also more likely to continue shared care compared to those seeing academic medical centre-family physician (OR = 4.30; 95%CI: 2.23–8.30). After excluding the 17 patients who were being seen by the polyclinic family physician, there was no difference in the success of the private family physicians when compared with academic medical centre-family physician with OR of 4.87 (95%CI: 1.70–13.92). When the polyclinic family physician was analysed as a third provider, the OR was 12.05 (95%CI: 3.43–42.35) compared to academic medical centre-family physician suggesting that shared care with the polyclinic family physician was at least as successful as with the private family physicians, if not better. However, the small sample size contributed to the wide confidence interval. There were no significant differences between patients who continued or failed shared care with respect to diagnosis and number of comorbidities, disease modifying anti-rheumatic drugs or other medications (Table 2).

### Interval between shared care initiation and first return appointment to Rheumatology Clinic among patients who completed one cycle of shared care

After adjusting for ethnicity and utilization status of medication courier service, older age, men, patients who had longer follow-up at Rheumatology Clinic and those with fewer medications were more likely to have a longer interval between initiation of shared care and return to Rheumatology Clinic (Table 3). Compared to men, female patients had a significantly higher chance of having a shorter time between being referred out for shared care and return to Rheumatology Clinic (HR = 2.18; 95%CI: 1.19–4.01). Increasing number of medications was significantly associated with shorter intervals between visits with hazard ratios increasing from 1.88 (95%CI: 1.10–3.22) to 3.09 (95%CI: 1.31–7.30) as the number of medications increased from between 5 and 8 medications to between 9 and 12 medications. In contrast, middle aged and elderly patients had a significant higher likelihood of a longer visit interval than those who were younger. Patients with a longer follow-up period were significantly more likely to have a longer visit interval with hazards 53–70% lower than those with ≤0.83 years (i.e. 10 months) of follow-up.

The academic medical centre-family physician saw 232 patients over the 29 months. Of these, 21 had shared care with a rheumatologist whereas 133 (57.3%) chose to remain with the family physician. The family physician initiated the return of 46 patients back to the rheumatologist (19.8%). The remaining 32 were discharged or defaulted follow-up.

## Discussion

Right siting is not well established in Singapore. Our endeavour is the first nationally that has been embedded into rheumatology practice in tertiary hospitals. Our results showed that private funding status and partnership with private family physician are predictors of persistence of shared care. Health care affordability constrained transfer of care into the community. Private laboratory monitoring was 1.3- to 2-fold more costly compared to subsidized charges in public hospitals. Private class patients paid 3-fold more to consult the rheumatologist as compared to the private family physician in shared care. The total cost to the private patient was therefore less in shared care. For subsidized patients, despite private family physicians agreement to match their consultation charges with the subsidized rate, this was insufficient incentive for patients to participate in shared care as hospital laboratory charges were lower. From the subsidized patient's perspective, he gets better value for money seeing a highly skilled hospital specialist who costs less due to the 50% government subsidy in easily accessible hospitals [10]. We can infer that patients who remained in shared care had continued stable disease as otherwise the shared care cycle would break due to the necessity to access the rheumatologist earlier than predicted. For those who preferred continuing hospital care, the default was shared care with the academic medical centre-family physician. This patient group is inherently reluctant for community care; moreover, they could disrupt shared care by asking for transfer of care back to the rheumatologist, hence biasing our findings of higher failure of academic medical centre-family physician. There were no significant differences between those who were successful or who failed shared care with respect to clinical or demographic characteristics, disease diagnosis and number of comorbidities and disease modifying anti-rheumatic drugs. Therefore, patients suitable for shared care are those who are privately funded and those with stable disease.

Intuitively, we thought that patients who had been followed up for years by a rheumatologist would be resistant to having their care either shared or transferred. Wee et al. found that follow-up of less than two years duration is a positive predictor of willingness to be discharged suggesting that stable patients could be discharged as soon as possible [12]. In contrast, longer duration of follow-up did not hamper shared care in our patients. Although the impetus for right siting should start within the first year, patients on longer follow-up were amenable to shared care.

Our research effort suggests that many subsidized patients, including those with a long-standing relationship with rheumatologists, would be willing to be managed by family physicians. However, this arrangement involved large out of pocket costs as they received subsidies for hospital care and were thus less likely to accept shared care. Despite our interventions to introduce subsidies (consultations and medications) for patients to see private family physicians, subsidized patients found it more affordable to remain within the hospital.

The efforts to encourage family physicians to take greater responsibility for the management of patients with musculoskeletal diseases were limited by health care financing arrangements in Singapore. Family physicians in polyclinics declined participation in shared care due to the perception of rheumatology being complex and challenging in the midst of their heavy workload and lack of familiarity with the use of disease modifying anti-rheumatic drugs. On average, polyclinic doctors saw twice the number of patients compared to private general practitioners (58 versus 30) [12].

Evidence suggests that strategies for care co-ordination often do not achieve their objectives [13]. Effecting system changes require substantial resources for process changes and change in mindset of patients, physicians and policy makers. Unless there are significant changes to Singapore's health care financing system, right siting efforts are destined to fail. Care co-ordination needs to be pursued primarily as a quality improvement strategy rather than one specifically aimed at reducing costs [14]. Moving outpatients to the community is unlikely to be cost effective [15]. Significant resources will be required to develop and grow primary care in Singapore which is in its infancy when compared to hospital care which, over decades, has been designed and financed as the major player of health care. Over time, as chronic disease care improves in the community, there will be a beneficial effect on health care outcomes and health care costs downstream [16].

International experience describes vertically integrated health systems with primary and tertiary components where funding and resources can be allocated to primary care as necessary, e.g. the decentralization of commissioning power to primary care practitioners in the United Kingdom. Reimbursement schemes in many countries do not reward innovations that support care co-ordination [17]. Previous right siting initiatives in Singapore have highlighted the negative impact to tertiary institutions’ financial position because revenue depends on patient volume [18]. Right siting, by moving patients out would therefore, reduce the amount of funding the hospital receives from the Ministry of Health. Those in the “driving seat” engineering health care services have to create a shared vision of a viable financial model to enable portability of subsidies to facilitate integration and move patients entrenched within hospital specialist clinics. Remuneration for more complex hospital work and right siting will incentivize the shift of patients from hospital to primary care. Similarly, stratifying work done by family physicians by complexity may incentivize chronic disease management to make it attractive for family physicians.

To improve population health, the cultural and structural divide in Singapore's health system has to be bridged. A broad, holistic concern for patients’ well-being and a narrow focus on the details of specific health conditions must synergize to provide comprehensive, accessible, affordable quality care. Health care professionals brought up to ‘favour’ tertiary care need reorientation to a mindset change. The politics of redistributing care are daunting, given most providers’ instinct to preserve the status quo and protect their turf. Partnerships across the previously sacrosanct boundaries of primary and hospital care are vital otherwise chronic disease management in primary care will fail. The annual “Essential Skills in Rheumatology” courses run by the Singapore Academy of Medicine and public hospitals continue to be avenues to provide rheumatology education to family physicians.

Patients’ cultural mindset must evolve from hospital to community care. We encountered difficulty convincing patients of the benefits of sharing care with a family physician whom they perceived as lacking the knowledge base to treat them. The question of whether quality of care is compromised arises when dealing with disease modifying anti-rheumatic drugs which have stringent safety monitoring requirements. In the United Kingdom, monitoring in primary care was at least as safe and acceptable to patients as secondary care-based services [19]. British, Dutch and Danish studies have shown that nursing consultations and different versions of shared care were not inferior to traditional rheumatologist review in controlling disease activity in patients with rheumatoid arthritis [20–24]. The safeguards put in place through recruitment of family physicians with family medicine training, attendance at Rheumatology Clinics, case manager's tracking of correspondence and blood tests were key to quality assurance.

Our clinical practice improvement project helped to systematize the workflow process to increase the numbers of patients right-sited. Information technology can aid the case manager's work in tracking and booking appointments with different health care providers. The national electronic health record was set up in November 2011 linking public health care institutions, as yet, only selected private health care providers are linked to this [25]. The academic medical centre-family physician clinic was a good indicator of how a family physician could manage patients in the community, as he was working within the hospital infrastructure. For family physicians, the case manager, in the absence of hospital infrastructure, delivered care integration through older modalities such as standardized forms, the telephone and fax [26]. Regardless, good communication and relationships build trust and social capital. Creating a new system of integrated care by defining the scope of service a provider can effectively deliver, together with identified partners and adopting common scheduling and other protocols help ensure that well-coordinated, multidisciplinary care is feasible.

## Conclusion

Our model of shared care, in the absence of organizational integration, had elements of integration at the micro level [27], such as a named case manager, systematic workflows, patient selection criteria and willing family physician partners. By coordinating services for individual patients and users to navigate the existing system, persistence of shared care suggested improved quality of rheumatology care. Future work to determine health-related quality of life would be valuable in assessing quality of care. Government subsidy for specialist outpatient care hindered the right siting of subsidized patients to private primary care. Compounding this barrier to right siting was the fact that doctors in polyclinics were significantly less likely to participate in shared care. Although we provided some reasonable evidence that it is possible to manage patients with musculoskeletal diseases in more appropriate, lower cost settings, it seems clear from our findings that the reach of these efforts will be extraordinarily limited without significant changes to the Singapore health care financing system.

## Figures and Tables

**Table 1.**
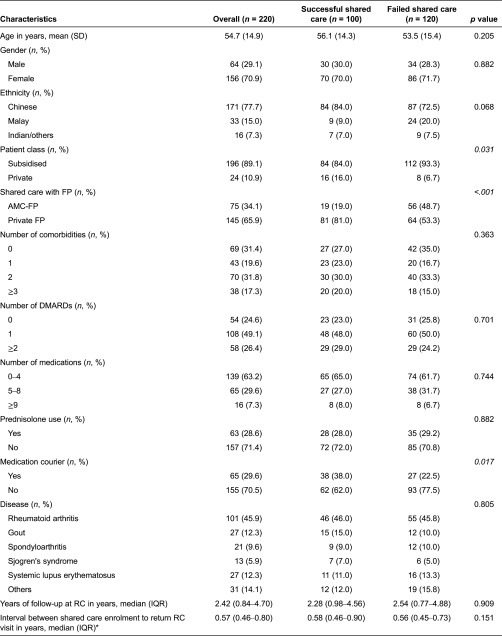
Patient demographics and clinical characteristics at enrolment into shared care

AMC, academic medical centre, DMARD, disease modifying anti-rheumatic drugs; FP, family physician; RC, Rheumatology Clinic; IQR: inter-quartile range

*Overall *p* value for categorical variables with more than two groups

**Table 2.**
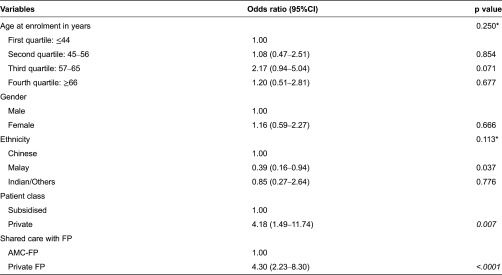
Multiple logistic regression for predictors of successful shared care

AMC, academic medical centre; FP, family physician

*Overall *p* value for categorical variables with more than two groups

**Table 3.**
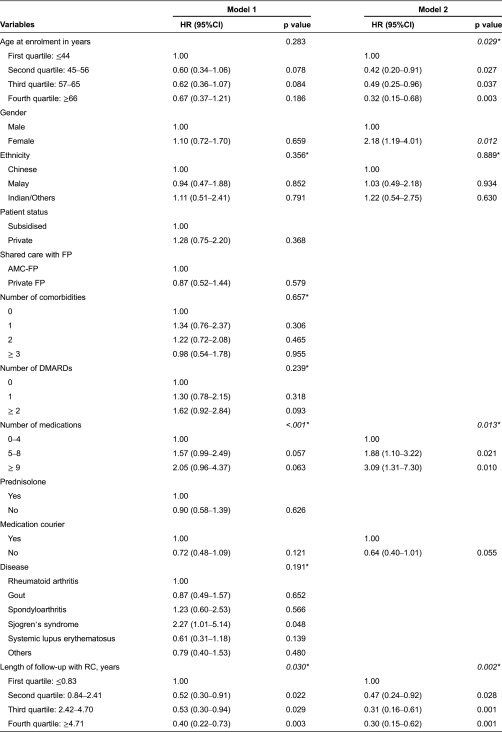
Cox regression analysis of covariates for interval between shared care enrolment and first return Rheumatology Clinic visit among patients who completed one cycle of shared care (*n* = 100)

Model 1: unadjusted

Model 2: adjusted for ethnicity and utilization status of medication courier service

CI, confidence interval; HR, hazard ratio; AMC, academic medical centre; FP, family physician; RC, Rheumatology Clinic

*Overall *p* value for categorical variables with more than two groups
